# Supplementary material for: Human gut microbes express functionally distinct endoglycosidases to metabolize the same N-glycan substrate
Source: Nat Commun. 2024 Jun 15;15:5123. doi: 10.1038/s41467-024-48802-3 (PMC11180146; doi:10.1038/s41467-024-48802-3)
Supplement: Supplementary file 1 — Supplementary Information [file 41467_2024_48802_MOESM1_ESM.pdf]

Human gut microbes express functionally distinct endoglycosidases to metabolize the same *N*-glycan substrate

Diego E. Sastre[1,*], Nazneen Sultana[1,x], Marcos V. A. S. Navarro[2,y], Maros Huliciak[1], Jonathan Du[1,z], Javier O. Cifuente[4], Maria Flowers[1], Xu Liu[1], Pete Lollar[3], Beatriz Trastoy[5,6], Marcelo E. Guerin[7] and Eric J. Sundberg[1,*]

[1] Department of Biochemistry, Emory University School of Medicine, Atlanta, GA 30322, USA
[2] Institute of Physics (IFSC-USP), University of São Paulo, São Carlos, SP, Brazil.
[3] Department of Pediatrics, Emory University School of Medicine, Atlanta, GA 30322, USA
[4] Instituto Biofisika (UPV/EHU, CSIC), University of the Basque Country, E-48940, Leioa, Spain
[5] Structural Glycoimmunology Laboratory, Biobizkaia Health Research Institute, Barakaldo, Bizkaia, 48903, Spain
[6] Ikerbasque, Basque Foundation for Science, 48009 Bilbao, Spain
[7] Structural Glycobiology Laboratory, Department of Structural and Molecular Biology; Molecular Biology Institute of Barcelona (IBMB), Spanish National Research Council (CSIC), Barcelona Science Park, c/Baldiri Reixac 4-8, Tower R, 08028 Barcelona, Catalonia, Spain.

*Address correspondence to: dsastre@emory.edu; eric.sundberg@emory.edu
[x]Present address: Structural Biochemistry Unit, National Institute of Dental and Craniofacial Research (NIDCR/NIH), Bethesda, MD, 20892, USA
[y]Present address: Center for Innovative Proteomics, Cornell University, Ithaca, NY, 14853, USA
[z]Present address: Sydney Pharmacy School, Faculty of Medicine and Health, The University of Sydney, Camperdown, NSW Australia.

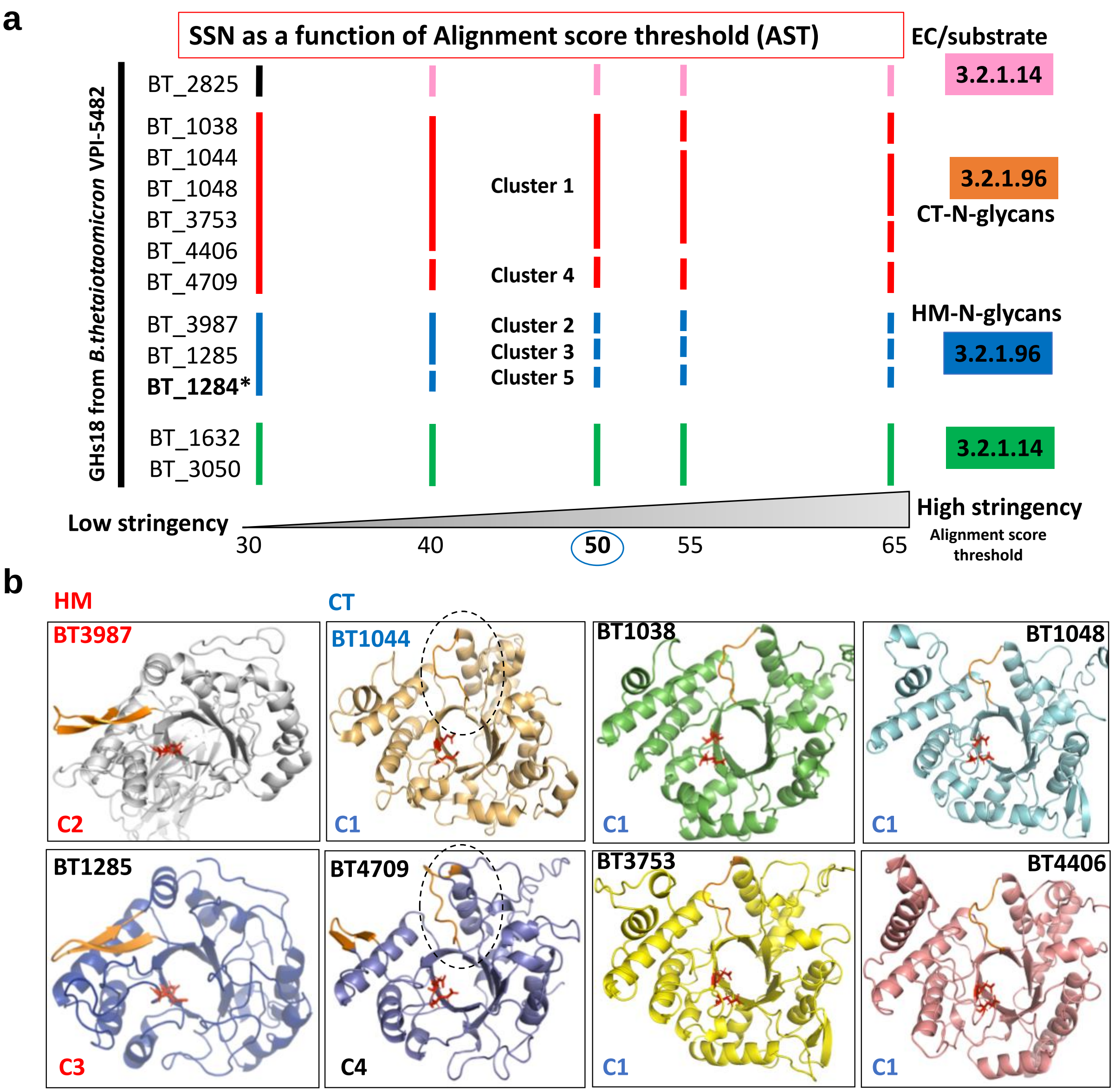

Supplementary Figure 1. Putative ENGases from *B. thetaiotaomicron* VPI-5482. a) Sensitivity analysis by testing different threshold values and assessing their impact on the resulting network topology. We used a range of AST from 30 to 65. Clusters from SSN (Figure 1) containing putative ENGases/endoglycosidases (GH18 family) from *B. thetaiotaomicron* VPI-5482 are indicated**. b**) Cartoon representation of GH18 domain (TIM-barrel fold) obtained by Alpha-fold prediction of BT1038, BT1048, BT4709, BT3753, BT4406 and BT1285. BT3987 (6TCV) and BT1044 (6Q64) were obtained from PDB databank (www.rcsb.org). A β-hairpin typical of HM-specific ENGases was orange colored.

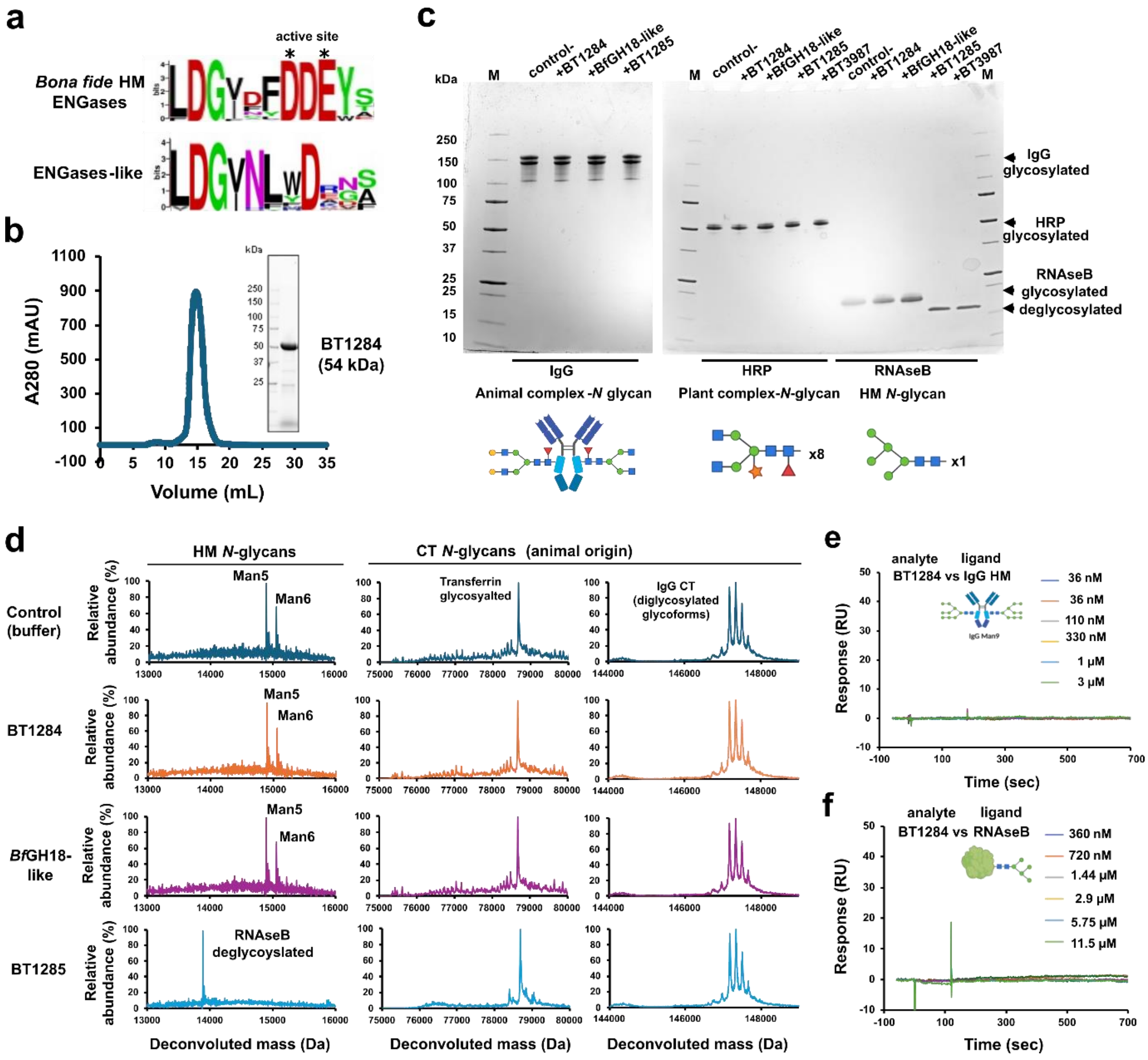

Supplementary Figure 2. ENGases-like in HM-PULs in Bacteroidales are catalytically impaired and are not binding to HM N-glycans. a) A sequence logo (obtained using WebLogo: https://weblogo.berkeley.edu/logo.cgi) represent the multiple sequence alignment of 350 protein sequences of bona fide ENGases and 85 protein sequences of ENGases-like around the putative active site including BT1284 encoded in the HM PUL 16 in *B. thetaiotaomicron*. **b)** Gel filtration analysis in Superdex S200 and SDS-PAGE of BT1284 purified fraction. **c**) SDS-PAGE gel for testing activity of ENGases like (BT1284 and BFGH18-like) and *bona fide* ENGases (BT1285 and BT3987) vs plant complex *N*-glycans from HRP, complex type *N*-glycans from animal (IgG CT) and HM-glycans from RNAseB. Assays were run in independent duplicates. **d**) LC-MS analysis of glycoprotein substrates vs ENGases-like. Relative amounts of the substrate and hydrolysis products were plotted using BioConfirm10.0 The spectra are representative of overninght incubation at 37 °C with PBS buffer (control), BT1284, 1284-like or BT1285 (HM ENGase) (50 nM) and substrates indicated (RNAseB, transferrin or IgG CT) at 2uM. Assays were run on technical triplicates. **e**) SPR Sensorgrams of BT124 (analyte) and IgG HM (ligand. **f**) SPR Sensorgrams of BT124 (analyte) and RNAseB (ligand). Range of analyte concentration used is indicated in the figure. Assays were run on independent duplicates. No binding was detected.

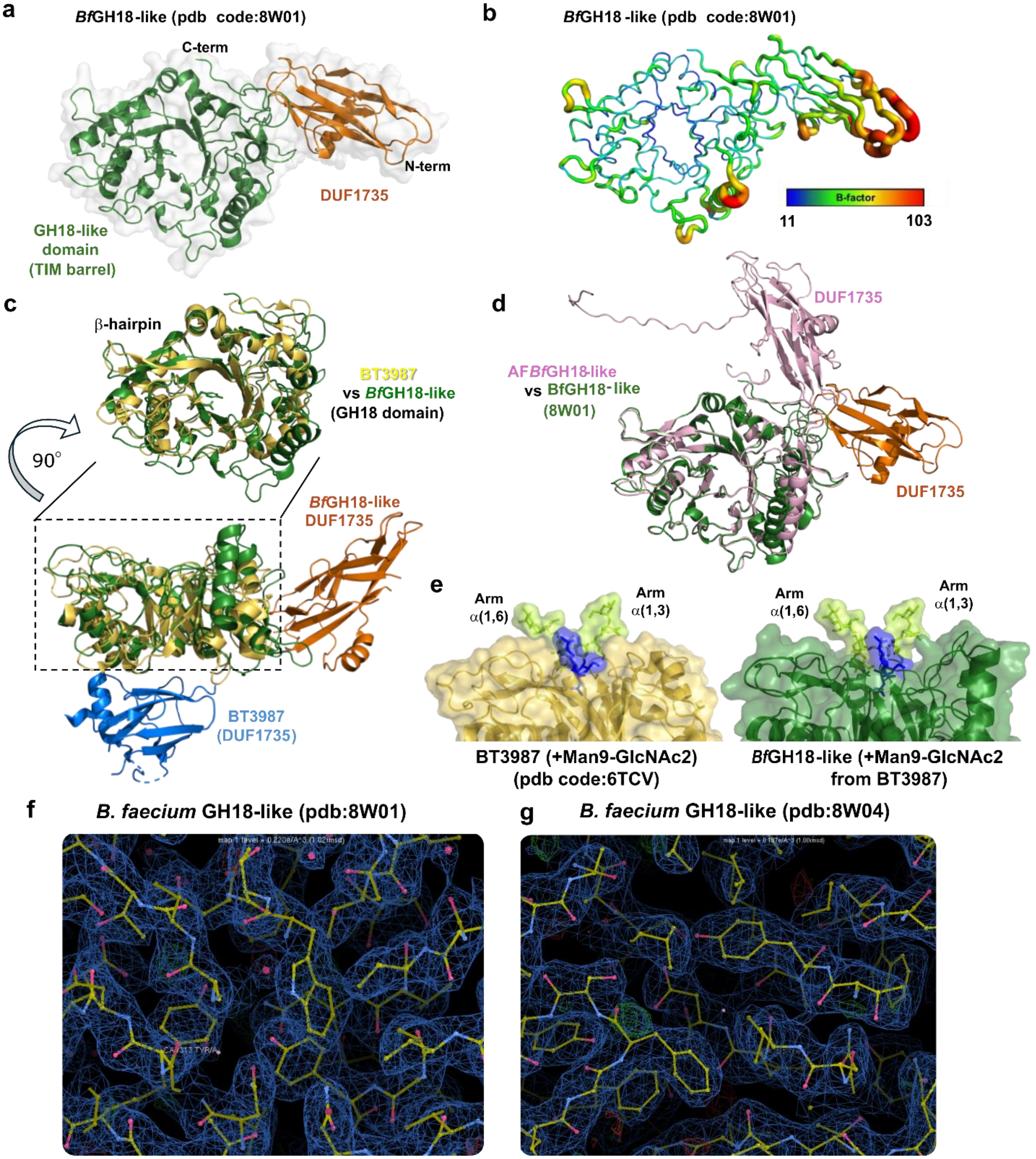

Supplementary Figure 3. Structural analysis of GH18-like (ENGase-like) from *B. faecium*. **a)** Cartoon representation of crystal structure of 1284-like apo (pdb code:8w01), showing the N-terminus β-sandwich domain (DUF1735) and the TIM barrel typical of GH18 enzymes. **b)** 1284-like crystal structure representation colored by B-factors. **c)** Superimposition of crystal structure of *Bf*GH18-like (green) and $BT3987_{D312A\text{-}E314L}$ (yellow) (pdb code:6TCV). DUF1735 domain of BT3987 (in blue) has different orientation than the similar domain in *Bf*GH18-like (orange). Y312 and E314 residues from inactive active site are represented as sticks. β-hairpin typical of HM-

specific ENGases is observed in both structures. **d)** Superimposition of AF model of 1284-like and 1284-like structure obtained in this study. DUF1735 is misplaced in the AF model. **e)** 1284-like surface representation with Man9-GlcNAc glycan from BT3987 (pdb code:6TCV) Glycan fitting shows no contact of 1284-like superficial residues with any HM N-glycan arm. **f** and **g**) Images of a portion of the electron density map 2*F*o-*F*c contoured at 1 σ level for each *Bf*GH18-like crystal structure.

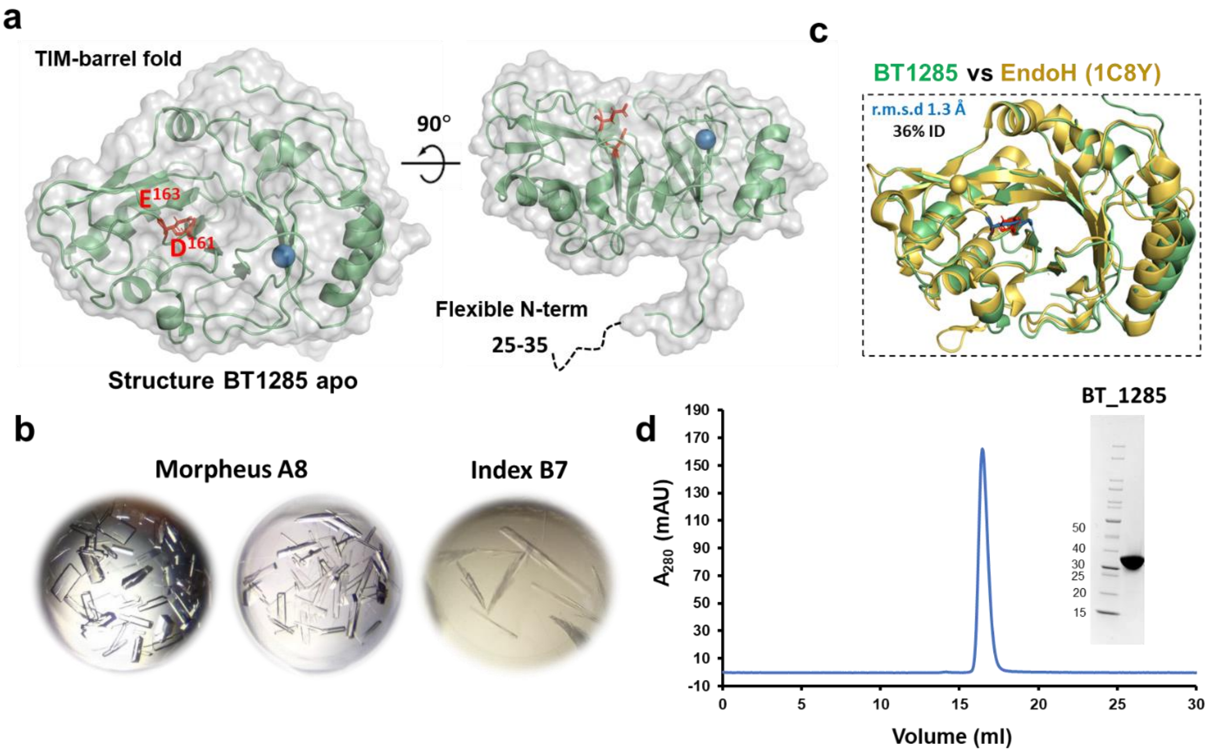

Supplementary Figure 4. Crystal structure of apo BT1285. a) Two views of cartoon representation of BT1285 wt apo structure at 1.1 Å resolution. N-terminus residues 25-35 are absent in the crystal structure may be due to high flexibility. **b**) Pictures of crystal in crystallization screening drops. **c**) Superimposition of BT1285 and EndoH (pdb: 1C8Y) structures revealed similar folding of TIM barrel domain (GH18 domain). **d**) Gel filtration analysis in Superdex S200 and SDS-PAGE of BT1285 purified fraction.

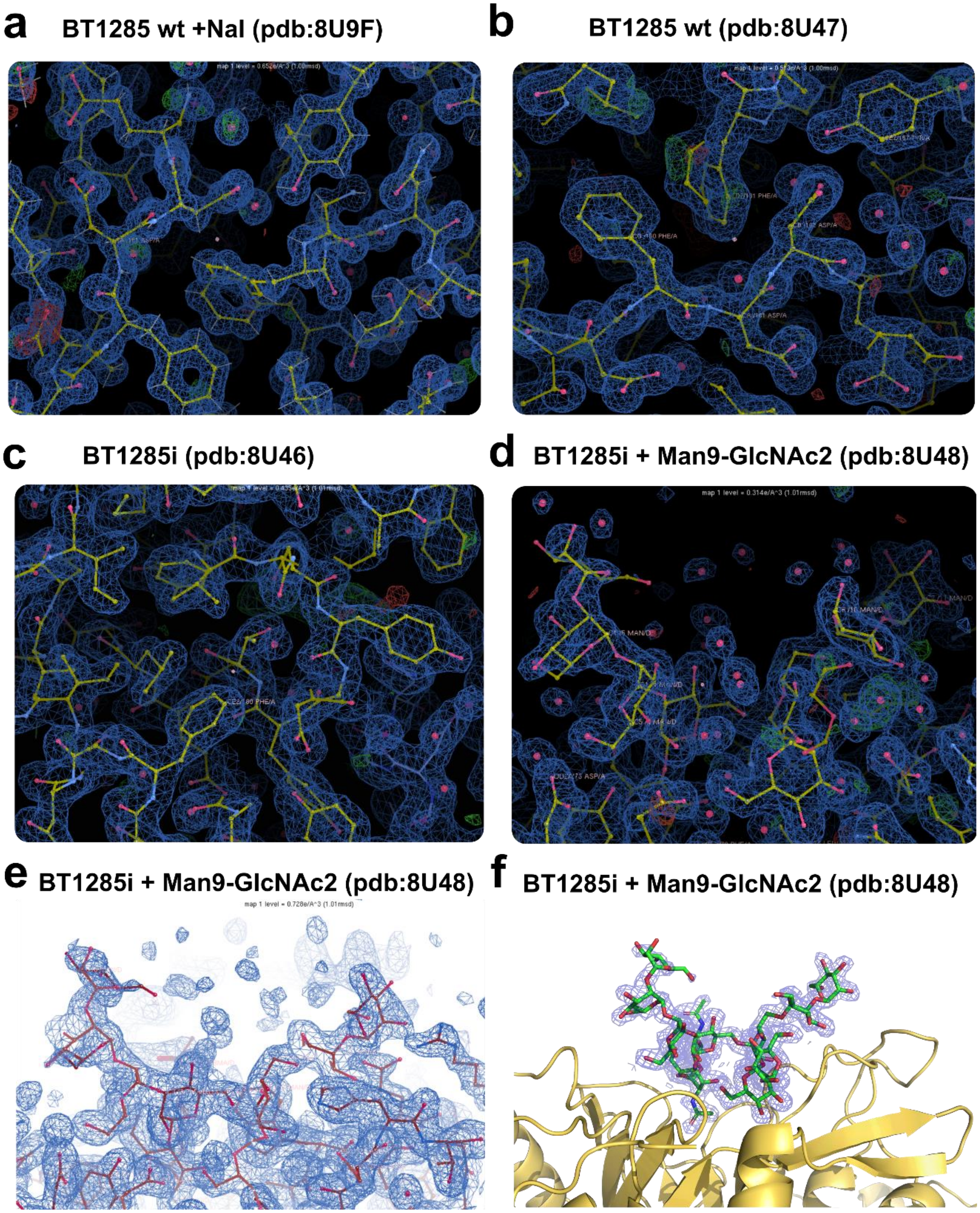

Supplementary Figure 5. Electron density maps of crystal structures of BT1285. (a-d) Images of a portion of the electron density maps 2*F*o-*F*c of crystal structures obtained in this study contoured at 1 σ using Coot. **e)** Composite omit map (2m*F*o-D*F*c) for the BT1285i-Man9-GlcNAc2 complex (PDB code 8U48) contoured at 1.0 σ is shown in blue. Composite omit map was generated using Composite omit map tool in Phenix. **f**) Electron density maps 2*F*o-*F*c and stick representation (green and red) of Man9-GlcNAc (in blue) observed in the cartoon representation (in yellow) of BT1285i-Man9-GlcNAc2 complex structure (PDB code 8U48).

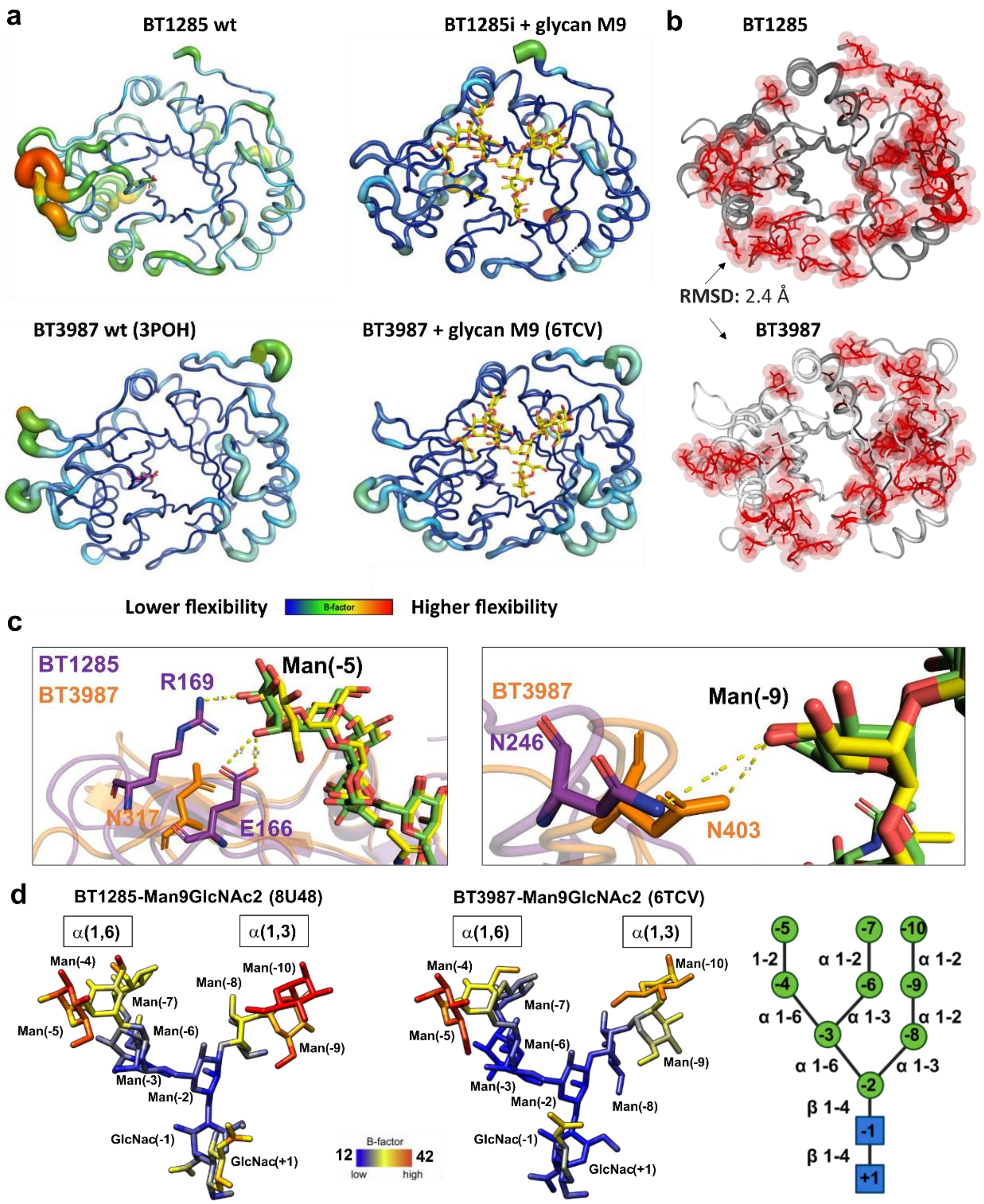

Supplementary Figure 6. Structural differences between BT1285 and BT3987 apo and glycan bound crystal structures. a) Cartoon representation of crystal structures of BT1285 and BT3987 apo and bound to Man9GlcNac2 (M9) colored by B-factors. **b**) Side chains differences (in red) between BT1285 and BT3987 ENGases are mostly located around glycan binding site as retrieved by 2StrucCompare (https://2struccompare.cryst.bbk.ac.uk/index.php). c) Close views of residues that interact with Man9 glycan on BT1285 but are not interacting with BT3987 (upper panel) and vice versa (lower panel). **d)** Relative B-factor representation of glycan substrates found in the crystal structures of BT1285 (PDB code 8U48) and BT3987 (PDB code 6TCV), indicating

flexibility level of HM N-glycan on the active site of BT1285 and BT3987 ENGases. The colors from blue to red indicate B factors values from blue (low flexibility) to red (high flexibility).

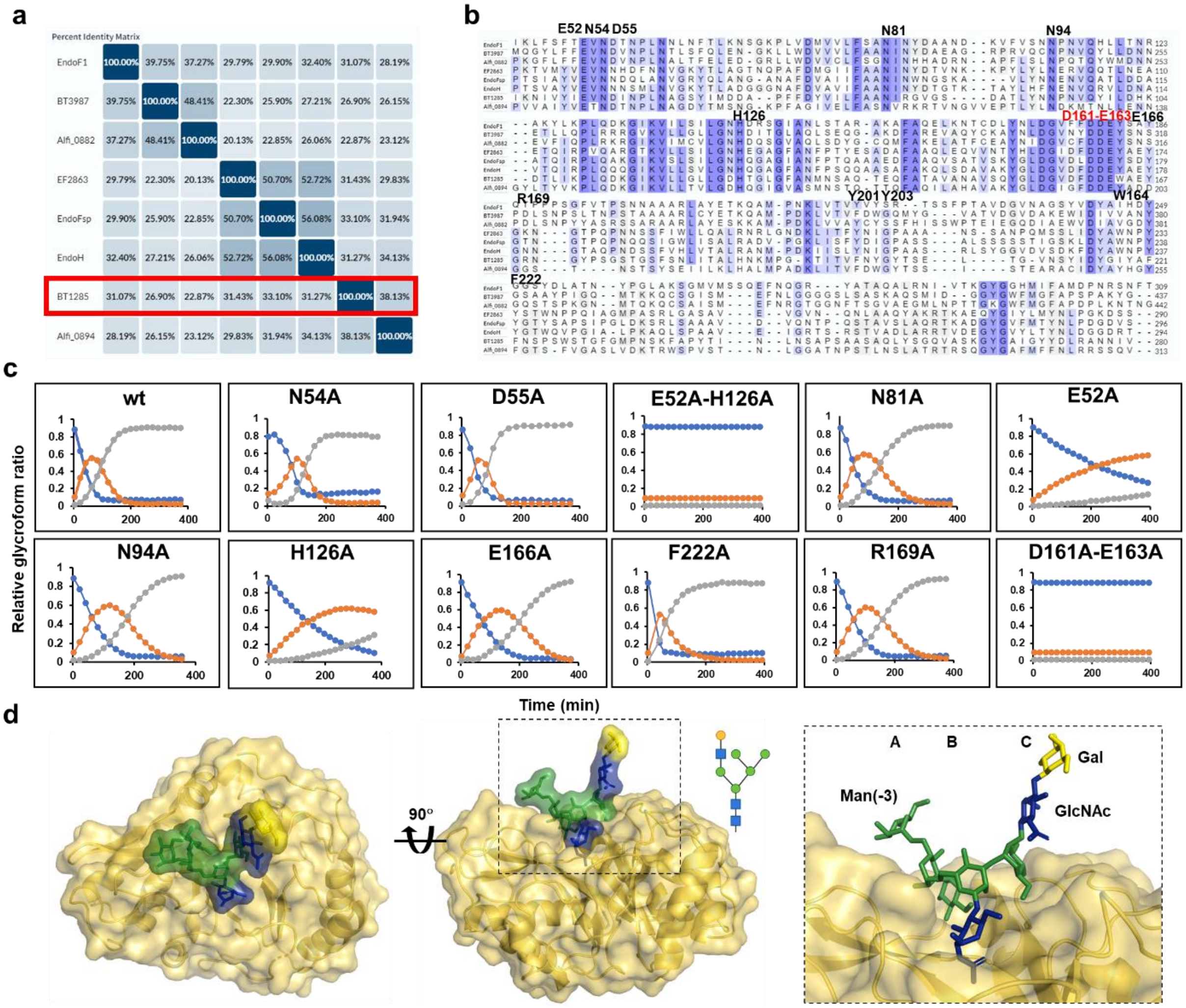

Supplementary Figure 7. Closest orthologs of BT1285 and their conserved residues. a) Percentage of identity matrix of characterized bacterial HM-specific ENGases. EF2863/*Ef*Endo18A (Q830C5_ENTFA), EndoH (EBAG_STRPL), EndoFsp (EBAG_FLAST), EndoF1 (EBA1_ELIME), BT1285 (Q8A889_BACTN), BT3987 (Q8A0N4_BACTN), Alfi_0882 (I3YJT2_ALIFI), Alfi_0894 (I3YJU4_ALIFI). **b**) Aminoacid sequence aligment of bacterial HM-specific ENGases indicating the residues of BT1285 mutagenized in this study. **c**) Kinetic traces of alanine scanning assays using IgG-HM as substrate. Curves are representatives of technical triplicates. **d**) Hybrid-type N-glycan (GalGlcNAcMan5GlcNAc) from BT3987 (PDB code: 7NWF) was superimposed into BT1285 substrate binding site. There is no contact with arm C of hybrid type *N*-glycans by BT1285.

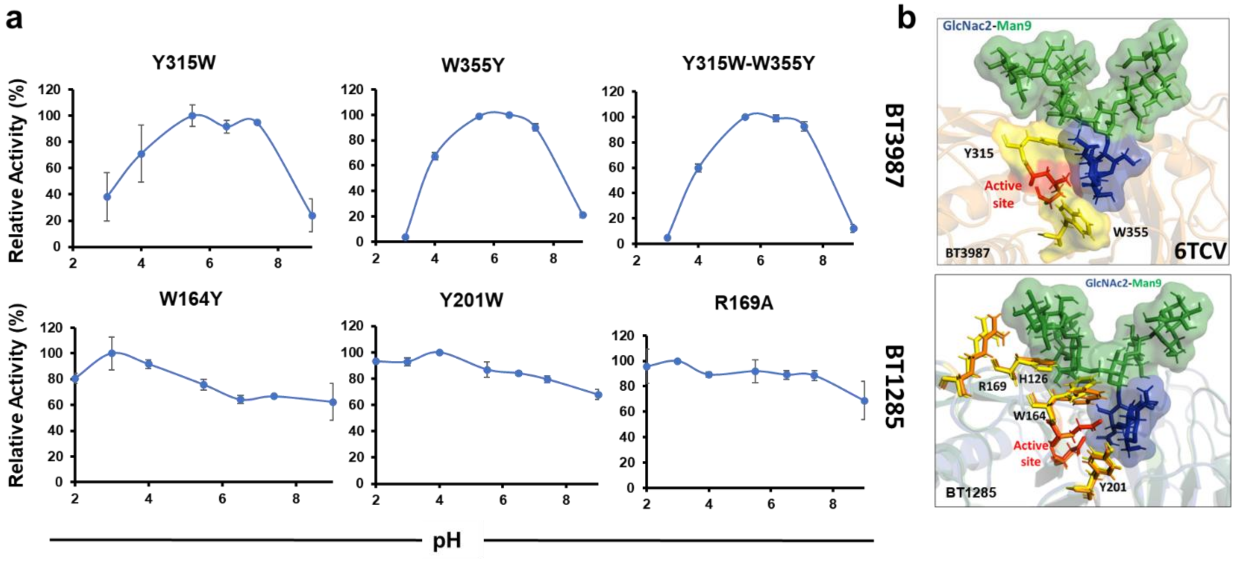

Supplementary Figure 8. pH dependence of BT1285 and BT3987 mutants around active site. **a**) Relative activity measurements of BT3987(upper panel) and B1285 (lower panel) enzymes using RNAseB as a substrate at different pH in the range of 2 to 9 at 23 °C. Assays were run on technical triplicates (n=3). Data are presented as mean values +/- SD. **b**) BT3987 + $Man_9GlcNAc_2$ active site (upper panel) and BT1285 + $Man_9GlcNac_2$ (lower panel). The unliganded form of BT1285 is superimposed into BT1285i + $Man_9GlcNAc_2$ in panel B.

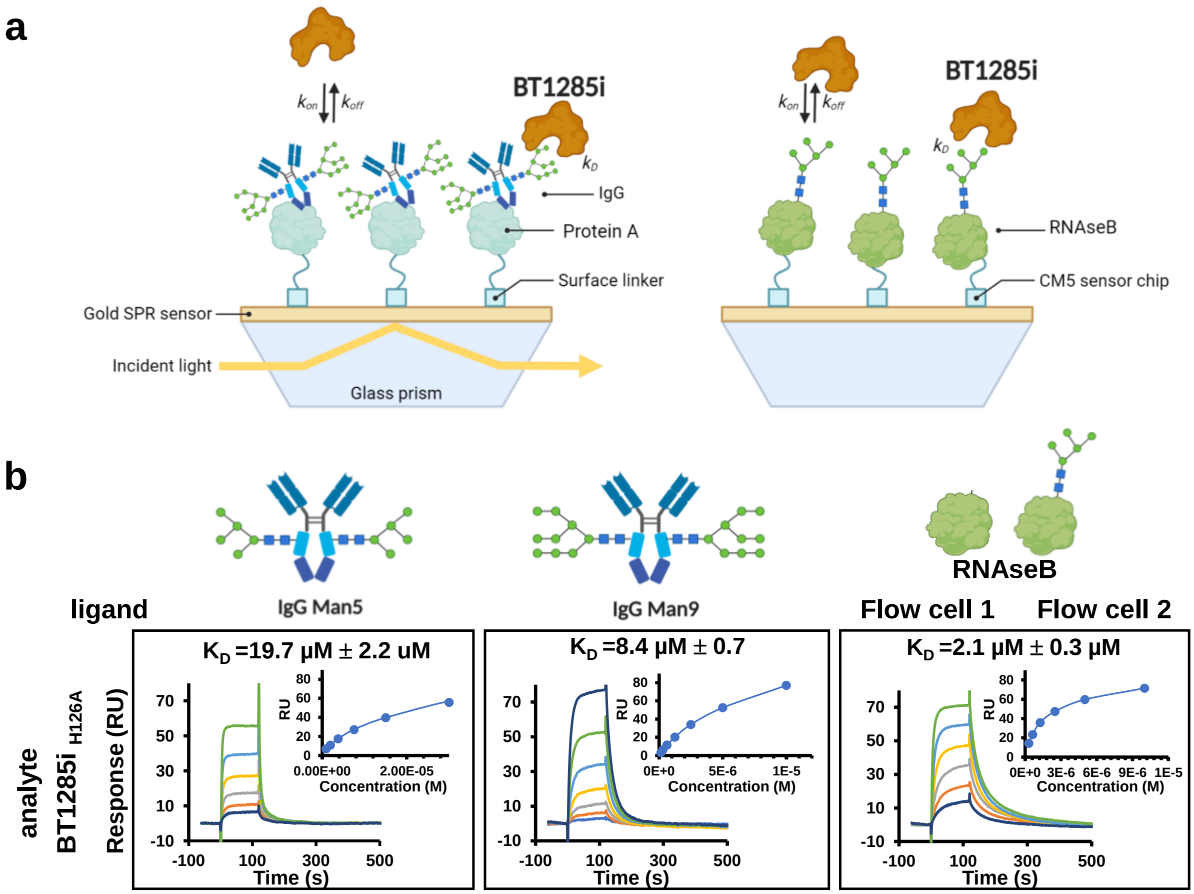

Supplementary Figure 9. Binding affinities of BT1285i H126A to HM-glycans. a) Schematic representation of Sensor Chips with ligands and analytes used in this study. **b**) SPR Sensorgrams and KD calculation of BT1285i$_{H126A}$ mutant. Range of analyte concentration used: (950 nM to 30 µM, for IgG-Man$_5$/Man$_5$ ligand); (150 nM to 10 µM, for IgG-Man$_9$/Man$_9$ ligand) and 312 nM to 10µM for RNAseB ligand). Assays were run on independent duplicates (n=2).

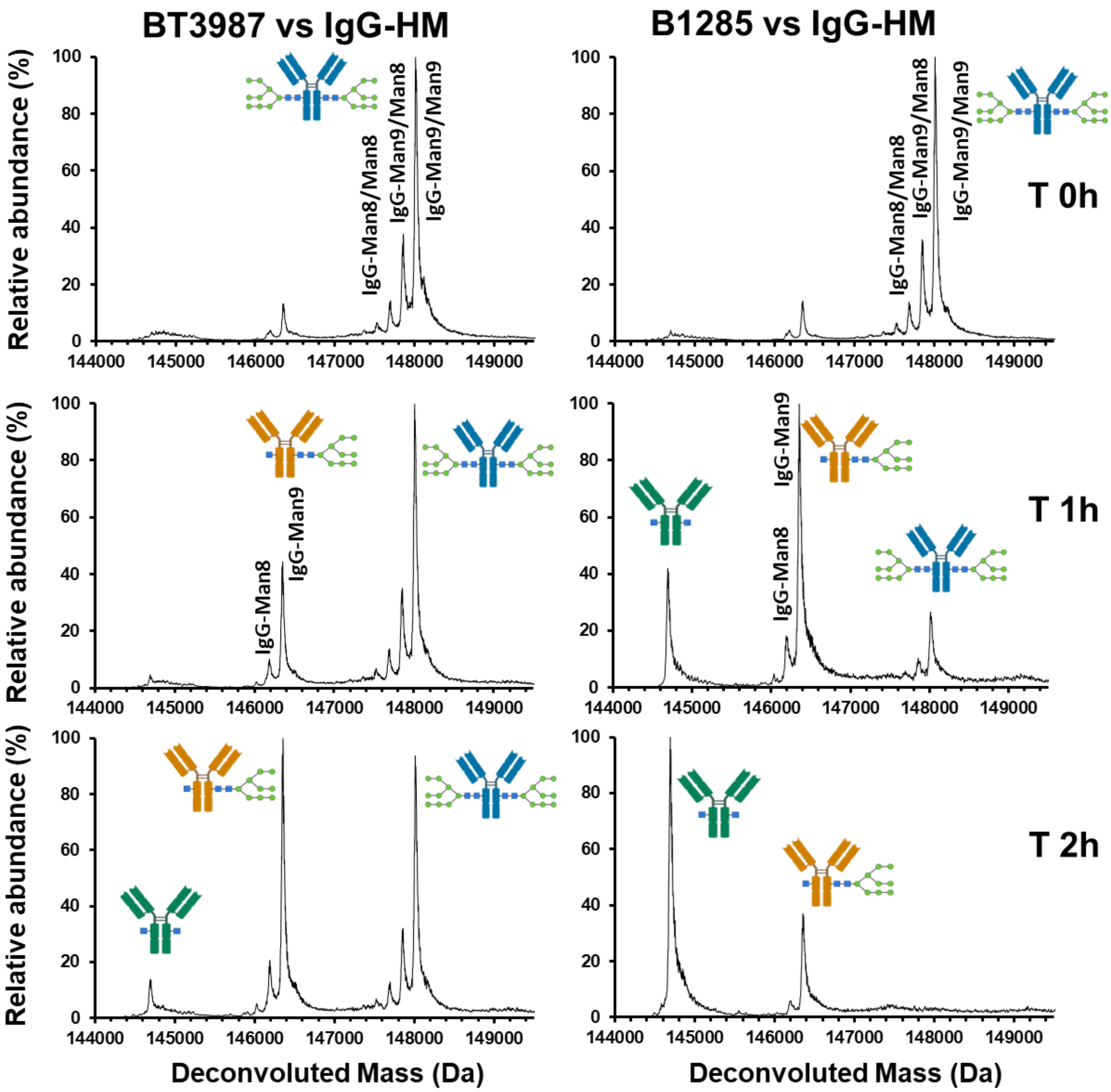

Supplementary Figure 10. Kinetic hydrolysis of BT1285 and BT3987 to HM- *N*-glycans on IgG. Relative amounts of the substrate and hydrolysis products were plotted using BioConfirm10.0 The spectra are representative of times 0, 1 and 2 hours of incubation between enzyme BT1285 or BT3987 (50 nM) and IgG-HM 2µM. The possible glycosylation species that exist include bi-glycosylated, N297-linked glycans on both IgG protomers (initial substrate), mono-glycosylated (an N297-linked glycan on only one IgG protome (intermediate), and deglycosylated (no Asn297-linked glycan on either protomer; the final product) are indicated in the graphic. Assays were run on triplicates.

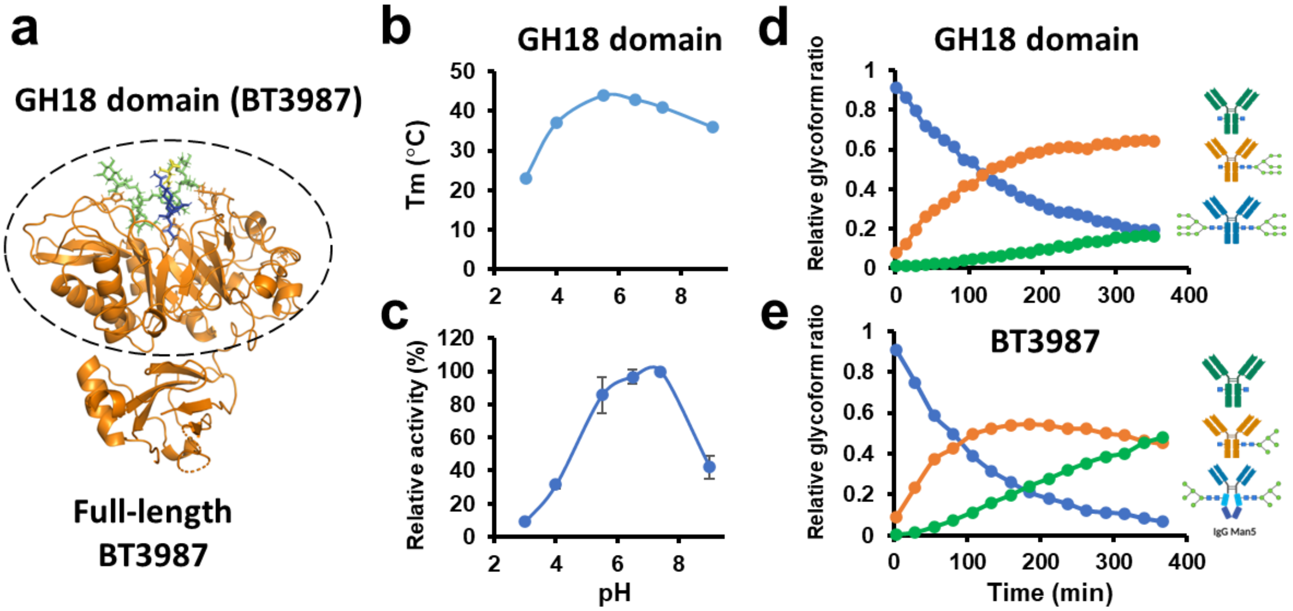

Supplementary Figure 11. GH18 domain of BT3987 has similar catalytic properties than full-length BT3987. a) Cartoon representation of the crystal structure of BT3987 (pdb 6TCV). The GH18 domain is indicated by the dash line. **b)** Melting point of GH18 domain of BT3987 determined by DSF at different pHs. **c)** ENGase activity of GH18 of BT3987 vs RNAseB substrate assayed at different pHs and incubated at 23° C. (n=3 technical replicates). Data are presented as mean values +/- SD. **d**) SEAK analysis of GH18 domain at pH 7.4 at 23° C using IgG-Man$_9$-Man$_9$ as substrate. **e**) Kinetic analysis of full-length BT3987 using IgG-Man$_5$/Man$_5$ as substrate at pH 7.4 at 37° C. Curves are representative of two experiments with similar results.

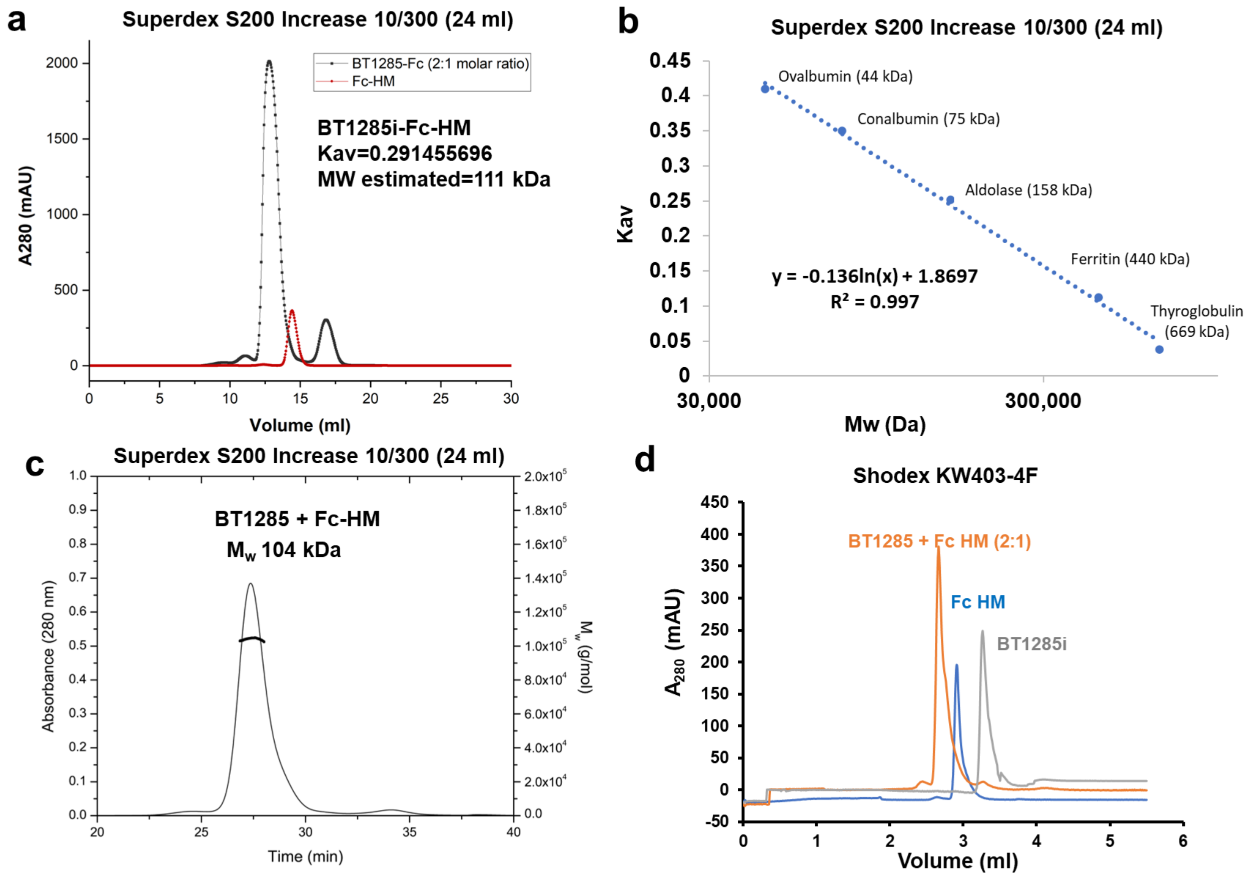

Supplementary Figure 12. BT1285i forms 2:1 stoichiometry complex with Fc-HM. a) Analytical Size Exclusion Chromatography (SEC) on Superdex S200 Increase 10/300 (24 mL). Chromatogram profile of Fc HM (red line) and BT1285i-FcHM 2:1 molar ratio complex (black line) in HBS buffer. **b**) Superdex S200 Increase 10/300 column calibration. **c**) SEC-MALS U.V chromatogram of BT1285i-Fc HM 2:1 molar ratio run on Superdex S-200. **d**) SEC of BT1285i (gray line), Fc HM (blue line) and BT1285i: Fc HM (2:1 molar ratio) on Shodex 404 KW403-4F column run in PBS buffer. Experiments were repeated independently two times with similar results.

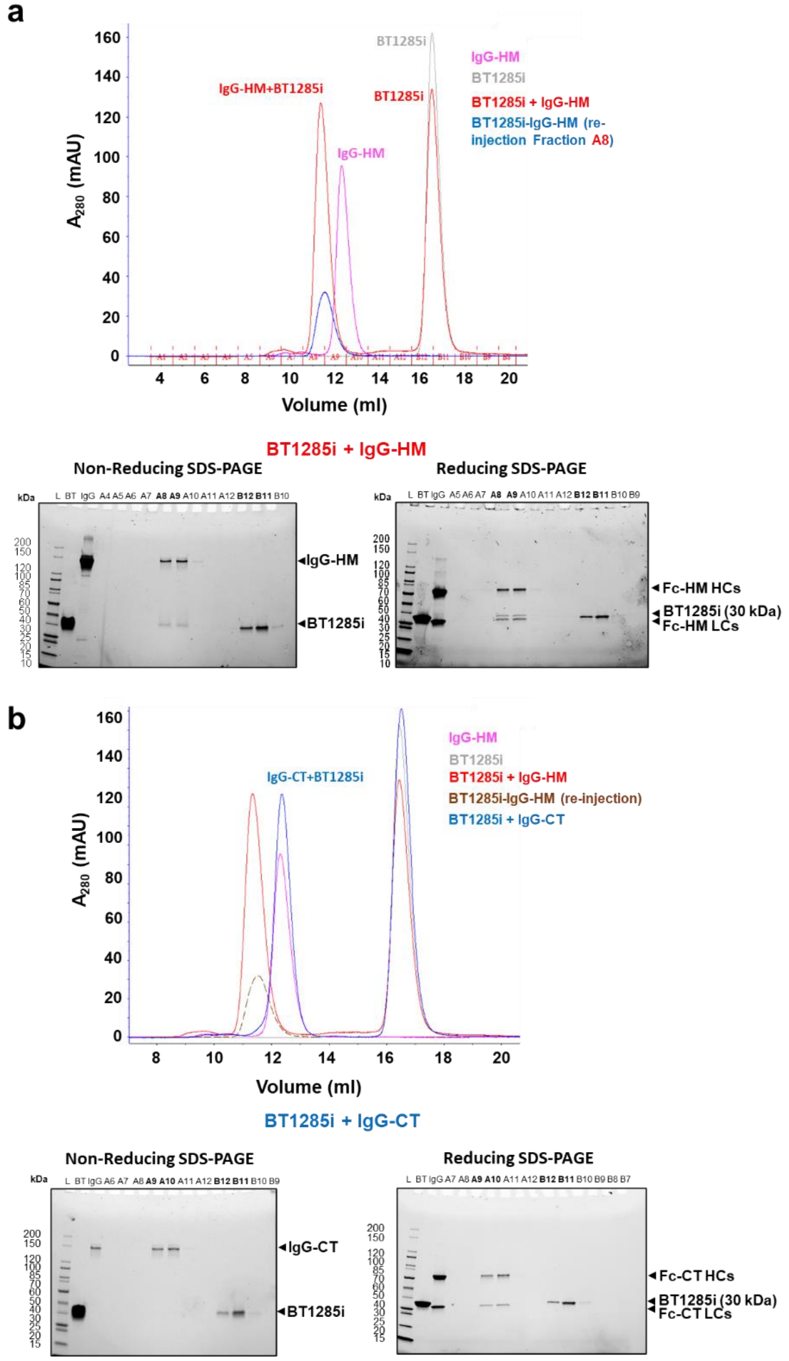

Supplementary Figure 13. BT1285i forms a stable complex with IgG1-HM but not with IgG-CT. IgG1-HM (**a**) and IgG1-CT (**b**) were incubated 5 min-RT with BT1285i in a 1:6 molar ratio, before injecting into a Superdex S200 Increase 10/300 column. Samples eluted (0.5 mL fractions) were collected and a fraction was loaded into stain free gels (Bio-Rad) for running in reducing conditions (with β-ME + 5 min-95 °C) or non-reducing conditions on SDS-PAGE. L (ladder), BT (BT1285i), IgG (IgG HM), HCs (heavy-chains), LCs (light-chains). Fraction A8 from the A panel was collected, and an aliquot was re-injected into the column (blue line and brown dash line in panel a and b, respectively). Experiments were repeated independently two times with similar results.

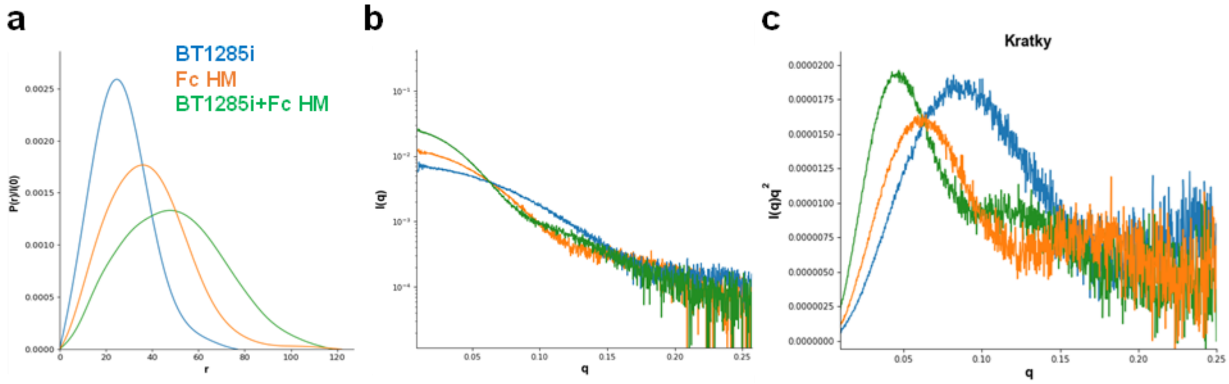

Supplementary Figure 14. SEC-SAXS analysis of the BT1285i-Fc-IgG1(HM) complex. (**a**) *P(r)* functions distributions of BT1285i (blue line), Fc-IgG1(HM) (orange line) and BT1285i-Fc-IgG1 (HM) complex (green line)**.** (**b**) SAXS scattering curve of BT1285i, Fc-IgG1(HM) and BT1285i-Fc-IgG1 (HM) complex. (**c**) Normalized Kratky plot of BT1285i, Fc-IgG1(HM) and BT1285i-Fc-IgG1 (HM) complex.

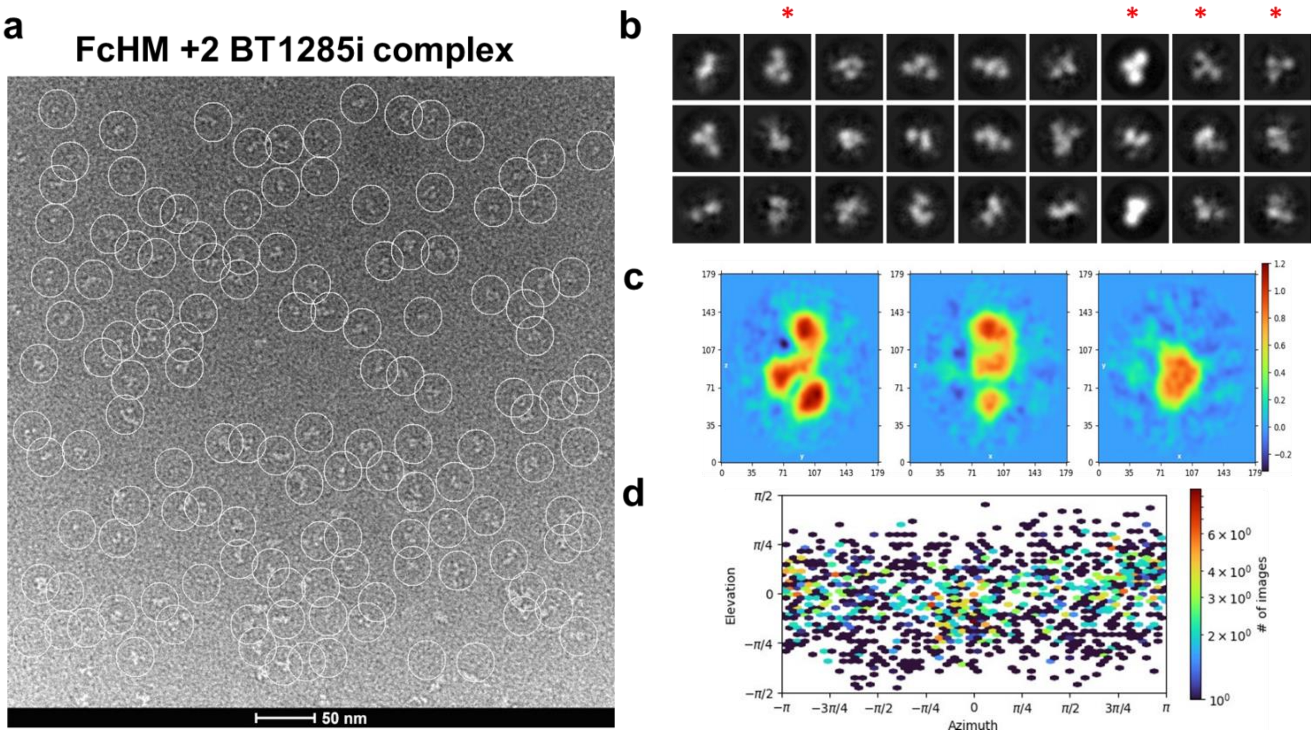

Supplementary Figure 15. ***Ab-initio*** **reconstruction of BT1285i-Fc HM by Negative-staining electron microscopy. a)** Micrograph and detail of particle picking results of the particles processed (ca.9000 picked particles). Particles are clear at glance. **b**) 2D classes used for ab-initio reconstruction. Several classes reveal particles larger than Fc and GH. Red asterisks represent some particles that were used to generate 10 ab-initio classes. **c** and **d**) The 10 models were set to refine against all particles (ca 9000 ptcls) for 3D classification. The class with interpretable features of a Fc+2 BT1285i complex and with higher number of particles (class 2 ca. 1200 particles) was selected for further processing. Other classes show lower quality but similar features, meanwhile other seems to represent FcHM-BT1285i 1:1 ratio.

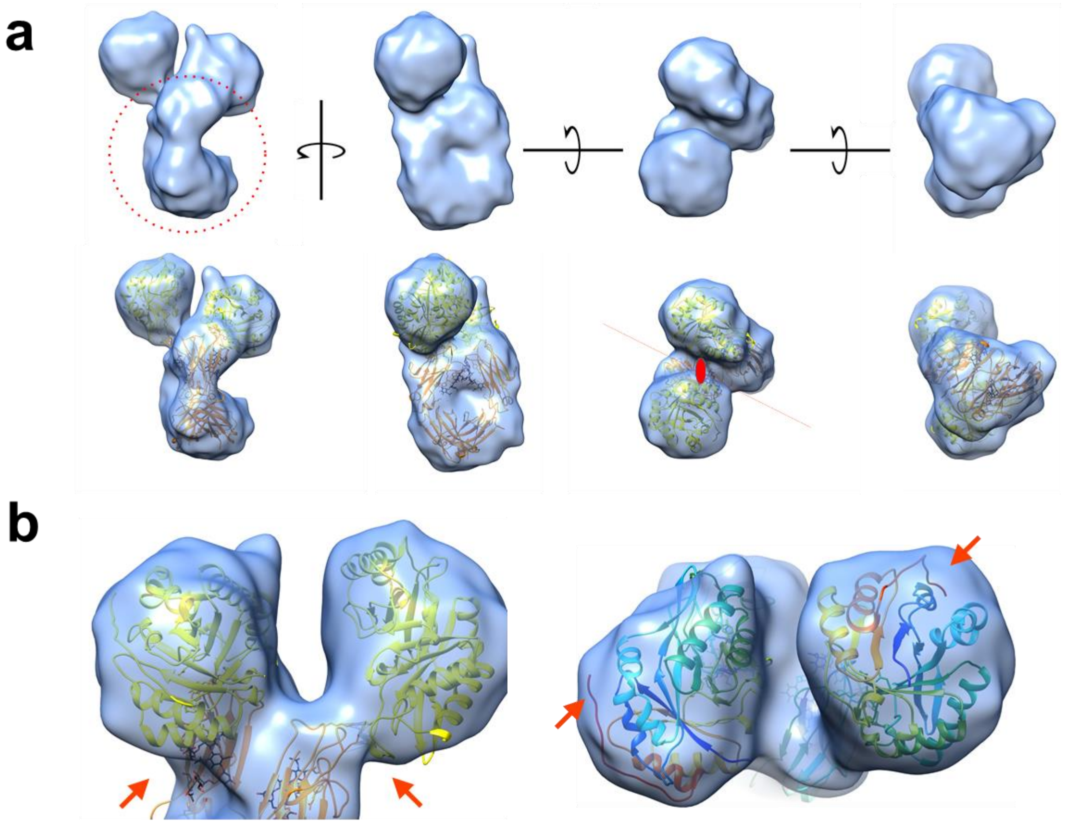

Supplementary Figure 16. 3D reconstruction of BT1285i-Fc HM complex 2:1 and fitting views. a) 90º rotations of refined C1 model display a good agreement with Fc (orange) and two GH bound on top of the CH2 domain. Note the Fc part of the reconstruction shows some flattening (a bit bent CH2-CH3) due to the negative stain (dashed circle). Top view shows a possible axis of C2 pseudo- symmetry that may indicate a higher symmetry in solution. **b)** Refined C1 model displays two different lobes for the GH fitting on top of the CH2 Fc (orange). Note the Fc-GH inter phases are different possibly due to the negative stain artifact or maybe the difference reflects two different binding modes.

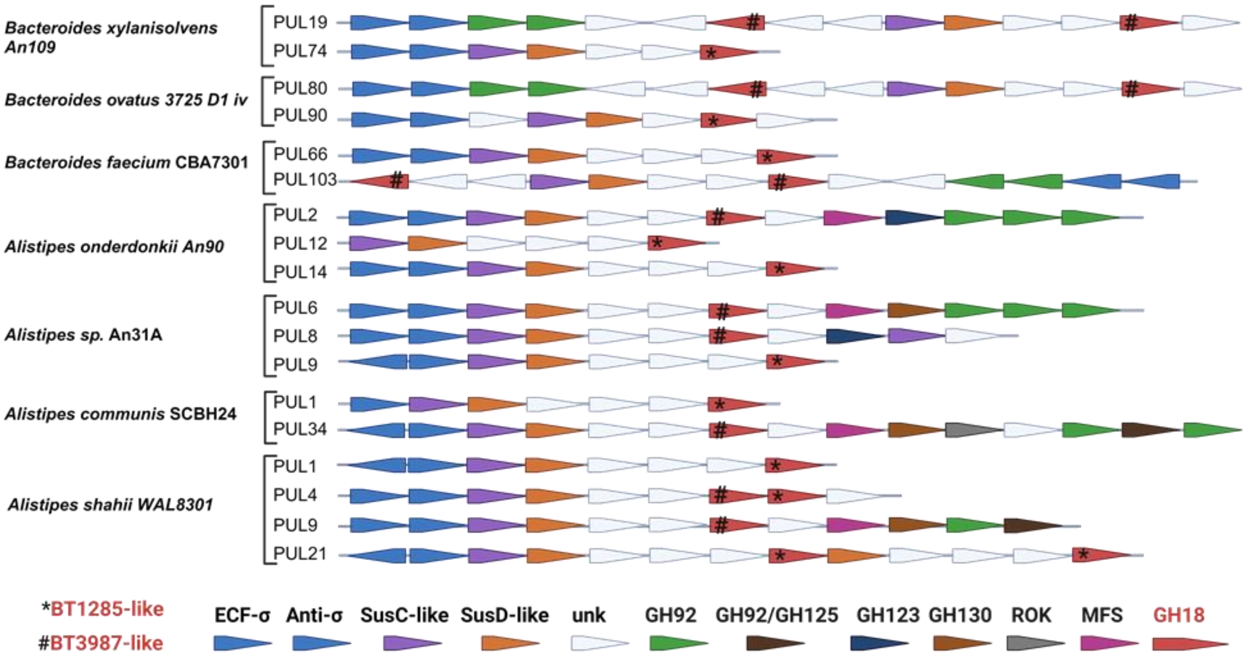

Supplementary Figure 17. Predicted Bacteroidetes PULs containing at least two different putative HM-specific ENGase. Predicted PULs were extracted from PULDB (http://www.cazy.org/PULDB/).

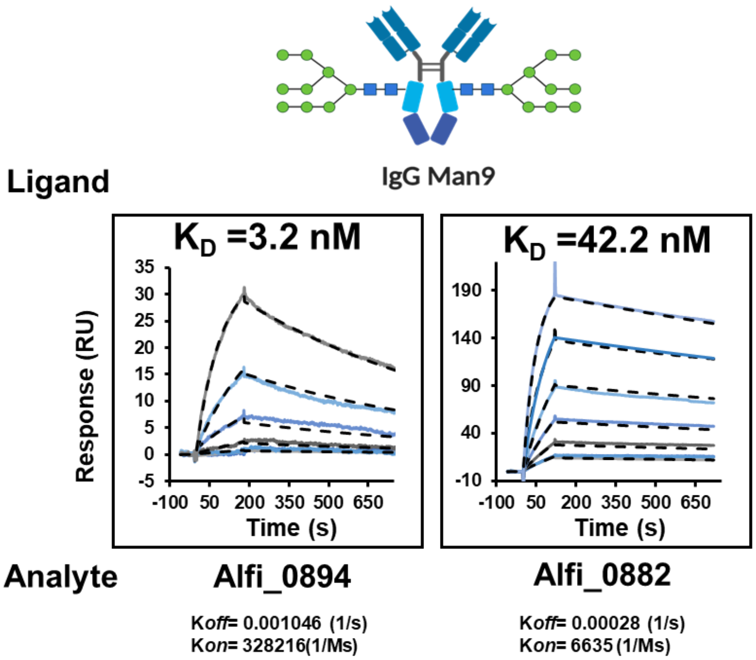

Supplementary Figure 18. Binding affinities of *A. finegoldii* HM processing ENGases to IgG HM-glycan. SPR Sensorgrams and K_D calculation of Alfi_0894i and Alfi_0882i. Range of analyte concentration used: Alfi_0894 (0.36 nM to 30 nM) and Alfi_0882 (93 nM to 3 µM), for IgG-Man_9 ligand. Assays were run on independent duplicates.

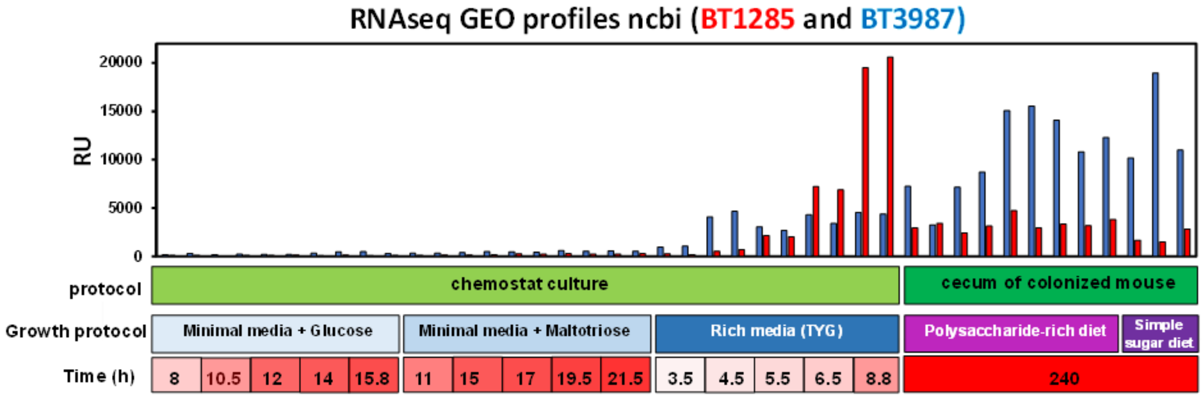

Supplementary Figure 19. Transcriptional profiles of BT1285 and BT3987 from *B. thetaiotamicron*. RNAseq GEO profiles obtained from ncbi (https://www.ncbi.nlm.nih.gov/geo/tools/profileGraph.cgi?ID=GDS1849:BT1285_at and https://www.ncbi.nlm.nih.gov/geo/tools/profileGraph.cgi?ID=GDS1849:BT3987_at). Each column represents the expression measurement extracted from the VALUE column of the original submitter Sample record. Expression values are considered arbitrary units. Despite the values of BT1285 and BT3987 being plotted in the same graph, the measurements are not comparable between them.

Supplementary Table 1. List of primers used in this study.

Primers name	Primer sequence (5'-3')
BT1285 BamHI F	GCTCACAAggatccATGCCAGTAAACCAATCAGATAATAATC
BT1285 XhoI R	CTTTAAAACctcgagTTAATAATTCTTTGGATAAGAATTAC
BT1285 D161A-E163A F	cgcgTGGGCTGAATACGGTAGA
BT1285 D161A-E163A R	tccgcAAAGTCTACACCATCTAATCC
BT1285-E52A-F	CGTTTATATTgcgGTAAATGATATCAATCCTC
BT1285-E52A-R	ATGTTTTTAATACCTGTGGC
BT1285-N54A-F	TATTGAAGTAgcgGATATCAATCCTCTTAATGC
BT1285-N54A-R	TAAACGATGTTTTTAATACCTG
BT1285-D55A-F	TGAAGTAAATgcgATCAATCCTCTTAATG
BT1285-D55A-R	ATATAAACGATGTTTTTAATACC
BT1285_N81A_F	GTTCGCTGCCgcgATAAGAGGTG
BT1285_N81A_R	AGAATTACATAATCGAAAAATGG
BT1285_N94A_F	TTTATATAACgcgCCAAATGTACAGTACATTTTGGAC
BT1285_N94A_R	GTAGCGTCTGAGCCTACA
BT1285_H126A_F	ATTAGGTGACgcgACAGGACTCGGATTC
BT1285_H126A_R	AGACCGAGTAAAACTTTG
BT1285_W164Y_F	TGACGACGAAtatGCTGAATACG
BT1285_W164Y_R	AAGTCTACACCATCTAATC
BT1285_E166A_F	CGAATGGGCTgcgTACGGTAGAAATG
BT1285_E166A_R	TCGTCAAAGTCTACACCATC
BT1285_R169A_F	TGAATACGGTgcgAATGGTTATCCATCTG
BT1285_R169A_R	GCCCATTCGTCGTCAAAG
BT1285_Y201W_F	TGTATTCAATtggGGCTACACAAGTG
BT1285_Y201W_R	GTAATCGTCTTACCTGGC
BT1285-Y201A-Y203A-F	cgcaACAAGTGAGCTTACAGGAG
BT1285-Y201A-Y203A-R	cccgcATTGAATACAGTAATCGTCTTAC
BT1285-F222A-F	TTACGCATTCgcgAACTCCCCATC
BT1285-F222A-R	ATGCCATAATCAATATAGC
BT3987_D312A-E314L	tctgTATTCAAACAGTCCTGATTTG
BT3987_D312A-E314L	tccgcATAATTTACTCCATCCAAGTTATATG
BT3987_GH18_A183_BamHI_F	GATATGGGATCCGCCGGTGATGCATATAAAGG
BT3987_GH18_XhoI_R	GGTTCTCGAGTTAAAGATCATCCGG
BT3987_Y315W_F	TGACGATGAGtggTCAAACAGTCC
BT3987_Y315W_R	TAATTTACTCCATCCAAGTTATATG
BT3987_W355Y_F	TGTATTTGATtatGGACAGATGTATGG
BT3987_W355Y_R	GTAACCAGTTTATCCGGC
Fc H268A/E269A/D270A/E272A_F	tcccgctGTGAAGTTCAATTGGTACGTG
Fc H268A/E269A/D270A/E272A_R	gcagcagcGGACACATCCACCACCAC
Alfi 0882i_D310A-E312A_F	TGCGTATTCCAACAGCCCGGAT
Alfi 0882i_D310A-E312A_R	TCCGCAAAGCACACGCCATCTATG
Alfi_0894i_D197A-E199A_F	TGCGTATGCTGATGATGGCGGC
Alfi_0894i_D197A-E199A_R	TCCGCAAACCCAATGCCATCCAG
BT1284 BamHI F	GTTTTATTGGGGGATCCATGGAAAATAACGATCTTAATATCG
BT1284 XhoI R	TATGAATGTCTCGAGTTAATAATCTTTAGGCATGAGAAG

Supplementary Table 2. Protein sequence of enzymes used in this study. Sequences that were removed from the construction appear underlined and in italic bold format.

**>BT1285 (Q8A889_BACTN)**<br>***MKNLYKILASSIAIFAFCACSQEE***MPVNQSDNNQSEVVTRSATGIKNIVYIEVNDINPLNAGSYIMDDAPFFDYVI<br>LFAANIRGVGSDATLYNNPNVQYILDHKDTLIKPLQDKGIKVLLGLLGDHTGLGFANMNSAQTEQFATAVANAVSQ<br>YGLDGVDFDDEWAEYGRNGYPSGSTGSFSNLITALHNKMPGKTITVFNYGYTSELTGVNSYIDYGIYAFFNSPSWS<br>TGFGMPNSKFAPYTINLNSAPSAASAQLYSGQVASKGYGAIGYYDLRANNIVSVLNGVAKGAFKSTCTYDGNSYPK<br>NY
**>BT3987 (Q8A0N4_BACTN)**<br>***MNMKYITSGLFAAMIVSSTAFLTSCA***DDLEVGKNIDESAYSGIYENNAYLRDGKSNLVSKVVELHGETYATTVKMG<br>LSKTPNTATSAKVKIDAAYLETYNKAHNTDFALYPQDLVTFANEGILTVNANTKSAEVEMTIRAGEGLQEDKTYAI<br>PVAISDQSSDITIKDEDAKHCIYLVKDMRNAGDAYKGEGVMQGYLFFEVNDVNPLNTLSFQLENGKLLWDVVVLFA<br>ANINYDAEAGRPRVQCNPNVQYLLDNNETLLQPLRRRGVKVLLGLLGNHDITGLAQLSEQGAKDFAREVAQYCKAY<br>NLDGVNYDDEYSNSPDLSNPSLTNPSTAAAARLCYETKQAMPDKLVTVFDWGQMYGVATVDGVDAKEWIDIVVANY<br>GSAAYPIGQMTKKQCSGISMEFNLGGGGSLSASKAQSMIDGGYGWFMGFAPSPAKYGSVFSRLQGGGEVLYGSNVA<br>APTIFYKKNDPTPYKYPDDL
**>GH18 domain_(A183-L476) (BT3987)**<br>AGDAYKGEGVMQGYLFFEVNDVNPLNTLSFQLENGKLLWDVVVLFAANINYDAEAGRPRVQCNPNVQYLLDNNETL<br>LQPLRRRGVKVLLGLLGNHDITGLAQLSEQGAKDFAREVAQYCKAYNLDGVNYDDEYSNSPDLSNPSLTNPSTAAA<br>ARLCYETKQAMPDKLVTVFDWGQMYGVATVDGVDAKEWIDIVVANYGSAAYPIGQMTKKQCSGISMEFNLGGGGSL<br>SASKAQSMIDGGYGWFMGFAPSPAKYGSVFSRLQGGGEVLYGSNVAAPTIFYKKNDPTPYKYPDDL
**>Alfi_0882 (I3YJT2_ALIFI)**<br>***MKTRYFKSGILAAAAALLCSAALVSC***TDDVTVGEWDGAGSIETGLNETGAMLQDLNSGKSNTTVELWKETYTADLR<br>LVLTKTPSEGFTARAKVDDSYDVGQYNKANGTNYTLYPADKVTFANDGLFAAAGRNVELTVGMTVHAAEGLVAGRG<br>YLIPVALEADGVILKESHCFYVVKDMTSMPTCYKGDDLPKGFLFFEVNDVNPLNALTFELEDGRLLWDVVCLFSGN<br>INHHADRNAPFLSLNPQTQYWMDNNEVFIQPLRKRGIKVIMCVLGNHDQSGVAQLSDYGCQMFAKELATFCEAYNI<br>DGVCFDDEYSNSPDLSNPYYASRSSARAARLAYESKKAMPDKLVVAYCYSSFHISSWPTEIEGQDIAEWVDIAVGD<br>YGQSTSPKGNMTQKQCSAISMEFNRGTGGNFTSGVAEGMLNPTTGKGWFMGFAPDPLKNTNGRVDKNAWRNIFVNR<br>LNKGPETLYGSPLKEPEHFYKFADTTRYNYPEDLPDTYSRPAQPEWPNY
**>Alfi_0894 (I3YJU4_ALIFI)**<br>***MKSEKCRKSSGRIINAPAKGGKSITNLNFCVMKNLFKLSMALFIGAAMFSC***QQAEEPTMDQGQAPETRAFGDTPVV<br>AIYVETNDTNPLNAGDYTMSNGKPFAGIVELFASNVRKRTVNGVVEPTLYLNDKMTNLLENNGYLTYVKPLQDKGI<br>KVLLTVLGDHQGIGVASMNSTQTTQFAQILAHAVAKYGLDGIGFDDEYADDGGSTNSTSYSEIILKLHALMPADKL<br>ITVFDWGYTSSISTEARACIDYAYHGYFGTSFVGASLVDKTRWSPVSTNLGGATNPSTLNSLATRTRSQGYGAFMF<br>FNLRRSSQVNPLNMFSATASGLYNGLTVTNANGNRAQDWTFVPAGYEINMDEVQ
**>BT1284 (Q8A890_BACTN)**<br>**MKQILKHILILAFAAGFIGTACE**NNDLNIDAGTFPETGGIGLSMGILQSDNYAMENPQINMDHASLSDQFHISLTE<br>PASQTGNYTVKVDESKVLDFNSKHGTSYPLYPTEYIDLGNSGKMTIEKGEQQSNSVSIAFKYDEAIEDSVIYVLPL<br>TVEENNSSPAMSSERKTLYYIINVWGMAPAEYNAIKKNFIQIAGVDPEFTNPLLLNKLYFESMSLSSPEVDYYNPF<br>DIINLQFATVKADDNLLPSLYLKDDLAYVLKKREKYIVPLQQLDHKVCLAIKGAGEGIGFSNLGEKEMMIFVERIK<br>QMIDIYHLDGVNLYDANFSYEESNENINYSNNLCKFVASLRDKLGNKIITYTQTSESPEGITNDANLKLGELLDYA<br>WCDQLNTIIDPWSTPEKWTRPIAGLNKEKWGALNTDIHMSSEQANILDQVIEMFTQPSLMITAGINHVFVVNRVDY<br>VSAGTESYAPTYMAYGAICNLCDMEKEYFVTGINSPNNQYLNIHDLLMPKDY
>**1284 -like from Bacteroides faecium (uniport ID:** A0A6H0KVH8**)**<br>**MKQILKLTMFLAAMAFVASCE**NNDIITDGGTFPETGGIDLTLGVLRSANYAEDNPLLEMDHKNVSDECMLTLTKPA<br>EQTITYTVGIDKTLVGAYNGKNGTNYTPFPGDVILTNEQLKLEKGKQESSKAHLEFTYDKNLASAIYLLPLIVKGT<br>SSNPAVSDSYQTIYYRINVWDEFAPAEYTTEPLVFTHIGYIDTENMNPLIANKLFYKLGREPHLSYVHAFSVINLL<br>TATVKYDQSGSMPEISYNKDISYVLGHAKKYIMPLQAQGHKVCLTIKGDGQGIGFSNLNATQSQKLVYDIRKCLEI<br>YGLDGVNLYDEDFSYKKEGDNLPSAANLCNFVTALRQAIDDKLITYAMTEESASGLDQSQNGIELGKIVDYAWTNQ<br>FNRLVNPWREDNPFGDDSQWKIAGLEQTKFGALTSTLKSLSQEEGELMEGSIFDNILDAGYMDLANVFVVNSIAKV<br>VAGVETQGATYLLWGALINYDVLQGINPELVPGLGKGGYLDIHSDLCPKDW

Supplementary Table 3. **Crystallographic data collection and refinement statistics for BT1285 wt +/-NaI, BT1285 inactive and inactive +HM-glycan.** *

	BT1285 wt + NaI (8U9F)	BT1285 wt (8U47)	BT1285 $_{D161A-E163A}$ (8U46)	BT1285 $_{D161A-E163A}$ + Man9GlcNAc2 (8U48)
**Data Collection**				
X ray source (Beamline)	SIRIUS LNLS-CNPEM (Manacá)	SER-CAT APS (ID-22)	SER-CAT APS (ID-22)	SER-CAT APS (ID-22)
Wavelength	0.977	1.000	1.000	1.000
Resolution (Å)	41.50 - 1.08 (1.14 - 1.08)	50.0 - 1.28 (1.33 - 1.28)	50.0 - 2.10 (2.18 - 2.10)	50.0 – 1.90 (1.93 – 1.90)
Space group	*P* 21 21 2	*P* 21 21 2	*P* 21 21 2	*P* 21
Unit cell (Å, °)	51.54 70.00 70.16 90 90 90	67.80 68.73 51.03 90 90 90	70.31 70.47 50.05 90 90 90	56.93 71.26 80.50 90 94.62 90
Total reflections	569201 (26747)	597060 (184932)	165972 (12788)	230828 (9095)
Unique reflections	191890 (17229)	53397 (4763)	14823 (1390)	49043 (2458)
Multiplicity	3.0 (1.6)	10.2 (4.4)	11.2 (9.2)	4.7 (3.7)
Completeness (%)	90.8 (50.5)	94.9 (68.5)	98.6 (95.5)	98.8 (98.3)
Mean I/sigma(I)	11.5 (2.8)	18.0 (1.3)	13.9 (3.4)	18.2 (4.0)
R-meas	6.6 (27.3)	0.116 (0.499)	0.188 (0.570)	0.238 (0.886)
CC1/2	0.997 (0.921)	1.000 (0.916)	0.985 (0.899)	0.987 (0.636)
**Refinement**				
Resolution (Å)	41.50 - 1.08	40.9 - 1.33	49.8 - 2.10	34.08 – 1.90
R-work	0.111	0.181	0.161	0.189
R-free	0.127	0.201	0.211	0.222
Number of atoms				
Protein	4440	2014	1997	4035
Solvent	357	240	168	276
Ligand	24	11	0	660
Average B-factor				
Protein atoms	10.62	17.36	25.54	22.72
Ligands	24.79	29.07		30.67
Solvent	23.96	26.81	32.36	35.03
Root mean square deviation				
Bond Lenghts (Å)	0.012	0.006	0.007	0.008
Angles (°)	1.333	0.896	0.838	0.846
Ramachandran statistics				
Residues in favoured region (%)	97.03	97.70	96.55	96.76
Residues in allowed region (%)	2.97	2.30	3.45	3.24
Residues in disallowed region (%)	0.00	0.00	0.00	0.00

#Statistics for the highest-resolution shell are shown in parentheses.

Supplementary Table 4. Crystallographic data collection and refinement statistics for *B. faecium* GH18-like (Uniprot ID: A0A6H0KVH8). *

	**1284-like (8W01)**	**1284-like (8W04)**
**Data Collection**		
**X-ray Source (Beamline)**	**SER-CAT APS (BM-22)**	**SER-CAT APS (BM-22)**
**Wavelength**	**1.000**	**1.000**
**Resolution (Å)**	**50.0 – 2.67 (2.72 – 2.67)**	**50.0 – 2.90 (2.95 – 2.90)**
**Space group**	**C 1 2 1**	**P 1 21 1**
**Unit cell (Å, °)**	**138.39 57.6 74.43 90 92.63 90**	**74.26 57.54 137.15 90 91.77 90**
**Total reflections**	**123036**	**182534**
**Unique reflections**	**16875 (1591)**	**25271 (2050)**
**Multiplicity**	**7.3 (5.5)**	**7.2 (6.8)**
**Completeness (%)**	**99.2 (98.9)**	**97.3 (79.9)**
**Mean I/sigma(I)**	**14.6 (2.1)**	**13.4 (2.3)**
**R-meas**	**0.198 (0.627)**	**0.207 (0.836)**
**CC1/2**	**0.958 (0.770)**	**0.972 (0.814)**
**Refinement**		
**Resolution (Å)**	**37.18 – 2.67**	**37.11 – 2.91**
**R-work**	**0.180**	**0.203**
**R-free**	**0.229**	**0.249**
**Number of atoms**		
**Protein**	**3545**	**7109**
**Solvent**	**134**	**0**
**Ligand**	**0**	**0**
**Average B-factor**		
**Protein atoms**	**35.11**	**43.97**
**Solvent**	**32.50**	**-**
**Root mean square deviation**		
**Bond Lenghts (Å)**	**0.004**	**0.003**
**Angles (°)**	**0.71**	**0.61**
**Ramachandran statistics**		
**Residues in favoured region (%)**	**96.22**	**96.89**
**Residues in allowed region (%)**	**3.56**	**2.78**
**Residues in disallowed region (%)**	**0.22**	**0.33**

[#]Statistics for the highest-resolution shell are shown in parentheses.

Supplementary Table 5. SAXS data collection and refinement parameters.

Data collection parameters	BT1285i-Fc-IgG1(HM)	BT1285i	Fc-IgG1 (HM)
**Instrument**	ID7A1(CHESS)	ID7A1(CHESS)	ID7A1(CHESS)
**Wavelength (Å)**	1.102	1.102	1.102
**S range (Å^{-1})**	0.0098 to 0.48	0.0098 to 0.48	0.0098 to 0.48
**Exposure time (s per frame)**	1	1	1
**Concentration (µM)**	325	85	240
**Temperature (K)**	277	277	277
***I*(0) (a.u.)[1] (from *P(r)*)**	0.0254	7.01 e-03	0.0133
***Rg* (Å) (from *P(r)*)**	38.44	21.12	29.8
***I*(0) (a.u.)[1] (from Guinier)**	0.0252	7.00 e-3	0.0132
***Rg* (Å) (from Guinier)**	38.0106	20.897	28.95
**D_{max} (Å)**	127	77	122
**Porod volume estimate (Å^3)**	151000	27000	69300
**Dry volume calculated from sequence (Å^3)[2]**	99925	36829	63118
**Molecular weight (kDa) (Qp)**	125.2	22.4	57.5
**Calculated MW from sequence (kDa)/ Discrepancy (%):**			
**Monomeric proteins**		30.4/26.3	55/4.3
**Enzyme:substrate (2:1)**	116/7.9		
**GNOM χ^2 value**	1.013	0.928	1.067
**Primary data reduction**	*BioXTAS RAW 2.1.1*	*BioXTAS RAW 2.1.1*	*BioXTAS RAW 2.1.1*
**Data processing**	*BioXTAS RAW 2.1.1*	*BioXTAS RAW 2.1.1*	*BioXTAS RAW 2.1.1*

[1]arbitrary units

[2] Dry volume determined using the server: http://biotools.nubic.northwestern.edu/proteincalc.html

[3] ATSAS
