# Supplementary material for: Human gut microbes express functionally distinct endoglycosidases to metabolize the same N-glycan substrate
Source: Nat Commun. 2024 Jun 15;15:5123. doi: 10.1038/s41467-024-48802-3 (PMC11180146; doi:10.1038/s41467-024-48802-3)
Supplement: Supplementary file 4 — Description of Additional Supplementary Files [file 41467_2024_48802_MOESM4_ESM.pdf]

File Name: Supplementary Data 1

Description: Analysis of the composition of each cluster from the Sequence Similarity Network.
